# Effects and Mechanisms of Tastants on the Gustatory-Salivary Reflex in Human Minor Salivary Glands

**DOI:** 10.1155/2018/3847075

**Published:** 2018-01-31

**Authors:** Shizuko Satoh-Kuriwada, Noriaki Shoji, Hiroyuki Miyake, Chiyo Watanabe, Takashi Sasano

**Affiliations:** Division of Oral Diagnosis, Department of Oral Medicine and Surgery, Tohoku University Graduate School of Dentistry, 4-1 Seiryo-machi, Aoba-ku, Sendai 980-8575, Japan

## Abstract

The effects and mechanisms of tastes on labial minor salivary gland (LMSG) secretion were investigated in 59 healthy individuals. Stimulation with each of the five basic tastes (i.e., sweet, salty, sour, bitter, and umami) onto the tongue induced LMSG secretion in a dose-dependent manner. Umami and sour tastes evoked greater secretion than did the other tastes. A synergistic effect of umami on LMSG secretion was recognized: a much greater increase in secretion was observed by a mixed solution of monosodium glutamate and inosine 5′-monophosphate than by each separate stimulation. Blood flow (BF) in the nearby labial mucosa also increased following stimulation by each taste except bitter. The BF change and LMSG secretion in each participant showed a significant positive correlation with all tastes, including bitter. Administration of cevimeline hydrochloride hydrate to the labial mucosa evoked a significant increase in both LMSG secretion and BF, while adrenaline, atropine, and pirenzepine decreased LMSG secretion and BF. The change in LMSG secretion and BF induced by each autonomic agent was significantly correlated in each participant. These results indicate that basic tastes can induce the gustatory-salivary reflex in human LMSGs and that parasympathetic regulation is involved in this mechanism.

## 1. Introduction

The minor salivary glands are vital for the maintenance of oral health because they secrete abundant mucin, which acts as a lubricant [1], and are involved in immunoactivity through secretion of immunoglobulin A [2]. Although the minor salivary glands contain less volume than the major salivary glands [3], they are widely distributed throughout the oral mucosa [4].

Eating is a strong stimulus for the secretion of saliva by the major salivary glands [5]. Large volumes of saliva are secreted before, during, and after eating via the gustatory-salivary reflex, masticatory-salivary reflex, olfactory-salivary reflex, and esophageal-salivary reflex. Parasympathetic efferent activities induced by taste stimuli have been shown to involve salivation and vasodilation in the major salivary glands [6]. However, the details of secretion mechanisms in the minor salivary glands remain unclear because of difficulties in collecting and quantifying the minute secretion volume from the minor salivary glands. We previously developed a new technique for measuring the minor salivary gland flow using a simple iodine-starch filter paper method [7] and demonstrated that the subjective feeling of dry mouth was more strongly related to a reduction in minor salivary gland flow than in whole salivary flow [8]. This finding suggests an important role of the minor salivary glands in xerostomia.

In the present study, we examined the effects of five basic taste stimuli (sweet, salty, sour, bitter, and umami) on reflex salivation in the human labial minor salivary glands (LMSGs). Specifically, we studied the synergistic effect of the umami taste on reflexive LMSG secretion because the combined umami taste of monosodium glutamate (MSG) and inosine 5′-monophosphate (IMP) is widely known to have a strong effect on taste perception as a characteristic feature of the umami taste [9]. Additionally, we investigated the nervous control of LMSG secretion using autonomic agents while monitoring the nearby blood flow (BF) in the labial mucosa where LMSG secretion was observed.

## 2. Materials and Methods

### 2.1. Participants and Exclusion Criteria

In total, 64 healthy participants were initially recruited from the students at Tohoku University and the residents and staff members at Tohoku University Hospital. Individuals with systemic disease (e.g., endocrine, infectious, or immunological disease or a history of chemotherapy and/or radiation therapy for head and neck cancer), who had been prescribed medications that could directly affect dry mouth, or who had a feeling of oral dryness were excluded. Individuals with hyposalivation identified by measuring the LMSG flow and those with a history of psychological problems were also excluded after careful interviews and psychological testing (Self-Rating Depression Scale) to avoid psychogenic oral dryness. Consequently, 56 individuals (average age, 31.2 ± 8.3 years; age range, 19–42 years; 44 men, 12 women) were finally included. These participants were divided into four groups to evaluate the LMSG responses to taste (*n* = 21), the synergistic effect of umami (*n* = 10), the relationship between LMSG secretion and the BF change where the LMSG are located (*n* = 14), and the involvement of the autonomic nervous system in LMSG secretion and the BF change (*n* = 11). This study was designed and conducted in complete accordance with the World Medical Association Declaration of Helsinki (http://www.wma.net) and was approved by the Ethics Committee of the Tohoku University Graduate School of Dentistry. Written consent was obtained from each participant after they had received an explanation of the purpose of the study.

### 2.2. Quantification of LMSG Secretion

The LMSG secretion responses to distilled water (DW), tastants, or autonomic agents were quantified using the iodine-starch filter paper method as previously described [7]. Briefly, a strip of test paper (3 × 1 cm, Filter paper 1; Toyo Roshi Kaisha, Ltd., Tokyo, Japan) painted with a solution of iodine in absolute alcohol and a fine starch powder mixed with castor oil was applied onto the lower lip for 2 min [7]. The blackened areas of each test paper, imprinted by the iodine-starch reaction, were scanned and digitized at 8 bits using a GT-9500 ART image scanner (Seiko Epson Corp., Nagoya, Japan) with the scanning resolution set at 144 dpi. The total area was measured using image analysis software (Scion Image Beta 4.02; Scion Corporation, Frederick, MD, USA). Each total area value was converted to a flow rate (*μ*L/cm^2^/min) using the calibration line *Y* = −0.084 + 24.992*X* (*Y*, area in mm^2^; *X*, volume in *μ*L) previously described [7].

### 2.3. BF Measurements in Labial Mucosa

BF changes in the lower labial mucosa, where quantification of the LMSG secretion was undertaken, were continuously monitored using reflection-mode laser Doppler flowmetry (SNF12007; Cyber Firm Med, Inc., Tokyo, Japan) before and after the administration of DW, tastants, or autonomic agents. During measurement of the BF, the participants were asked to keep their mouths open, and the lower lip was everted using an angle widener. The test areas were isolated with rolled gauze and then dried with a cotton gauze pad immediately before the recording was performed. A sensor probe was firmly anchored to the angle widener with surgical tape, and the tip of the probe was kept at a distance of 0.5 mm from the lip surface. All recordings were electrically calibrated to zero BF. Laser Doppler signals from the lower labial mucosa were continuously monitored, together with the systemic blood pressure (BP) (Finometer; Finapres Medical Systems, Amsterdam, Netherlands). The outputs from the flowmeter and BP monitor were recorded on a multichannel chart recorder (Recti-Horiz-8K; NEC San-ei, Tokyo, Japan).

### 2.4. Taste Stimulation

#### 2.4.1. Five Basic Tastes

Five well-established taste substances were used. For the four basic tastes (sweet, salty, sour, and bitter), ready-made test solutions of Taste Disc™ (Sanwa Chemical Co., Ltd., Nagoya, Japan) were used. Each concentration of the four basic tastes was as follows:

- Sweet (sucrose): 8.7 mM (No. 1: S1), 73.0 mM (No. 2: S2), 292.1 mM (No. 3: S3), 584.2 mM (No. 4: S4), and 2337.1 mM (No. 5: S5)
- Salty (NaCl): 51.3 mM (No. 1: N1), 213.8 mM (No. 2: N2), 855.5 mM (No. 3: N3), 1711.1 mM (No. 4: N4), and 3422.3 mM (No. 5: N5)
- Sour (tartaric acid): 1.3 mM (No. 1: T1), 13.3 mM (No. 2: T2), 133.2 mM (No. 3: T3), 266.5 mM (No. 4: T4), and 533.0 mM (No. 5: T5)
- Bitter (quinine): 0.03 mM (No. 1: Q1), 0.5 mM (No. 2: Q2), 2.5 mM (No. 3: Q3), 12.6 mM (No. 4: Q4), and 100.7 mM (No. 5: Q5)

For umami taste, MSG aqueous solution previously developed for an umami taste sensitivity test [10] was used. The concentrations of the umami taste were 1 mM (No. 1: G1), 5 mM (No. 2: G2), 10 mM (No. 3: G3), 50 mM (No. 4: G4), and 100 mM (No. 5: G5). Each taste number (Nos. 1–5) was set up so that the intensity of the participant's perception of the taste was equivalent in spite of the different taste qualities based on previously reported data of the Taste Disc [11] and our previously described findings regarding umami [10]. Thus, the intensity of perception of the same taste number (Nos. 1–5) was equal among the five tastes.

A 5 mm diameter cotton ball containing 50 *μ*L of each taste solution or DW was applied onto the posterior tongue for 2 min, and the LMSG secretion was then measured. The participants were asked to rinse their mouth with water for at least 15 min between each taste stimulation. The next taste stimulation was applied after the measurement value had returned to the baseline level. All participants (*n* = 21) were asked to refrain from eating or drinking (except water), smoking, and brushing their teeth for at least 3 h before testing.

#### 2.4.2. Combined Umami Tastes of MSG and IMP

To investigate the well-known synergistic effect of combined umami tastes on LMSG secretion, 5 mM of MSG aqueous solution, 5 mM of IMP aqueous solution, and a solution containing a mixture of the two (5 mM MSG and 5 mM IMP) were prepared. Combined umami tastes that have been shown to evoke a synergistic effect [9] were made using an aqueous solution containing 10 mM MSG and 10 mM IMP. Changes in LMSG secretion were quantified following each administration of MSG, IMP, or MSG + IMP solution onto the posterior tongue of 10 participants. The procedure was similar to the above-described experiment involving the five basic tastes.

### 2.5. Relationship between LMSG Secretion and Nearby BF Change following Taste Stimulation

The LMSG secretion and nearby BF changes in the labial mucosa following application of the highest concentration (No. 5) of each of the five basic taste solutions onto the posterior tongue were observed for 14 participants. The LMSG secretion was first measured, and then the BF change to the tastant was measured until the BF had recovered to the prestimulus value.

### 2.6. Use of Autonomic Agents

The following four autonomic agents were prepared:

- 0.1% adrenaline: a sympathomimetic agent (Adrenaline Injection 0.1%; Terumo Corporation, Tokyo, Japan)
- 3% cevimeline hydrochloride aqueous solution: a muscarinic (M3) receptor agonist (Saligren® capsule 30 mg; Nippon Kayaku, Tokyo, Japan)
- 1% atropine sulfate hydrate: a cholinergic blocking agent (atropine ophthalmic solution 1%; Nitten Pharmaceutical Co., Ltd., Nagoya, Japan)
- 2.5% pirenzepine hydrochloride aqueous solution: a muscarinic (M1) receptor antagonist (pirenzepine hydrochloride tablets 25 mg; Nichi-Iko Pharmaceutical Co., Ltd., Toyoma, Japan)

The concentration of each autonomic agent was based on the manufacturer's medical package insert for clinical use. The LMSG flow rate and BF were measured following application of a 3 × 1 cm filter paper soaked in 50 *μ*L of each agent or DW on the labial mucosa for 5 min in 11 participants. Stimulation with the next agent was applied after the measurement values had returned to the baseline level. The participants were asked to rinse their mouth with water, and an interval of at least 30 min was set between each stimulation.

### 2.7. Data Analysis

The LMSG secretion after the administration of DW, tastants, or autonomic agents is presented as a percentage of the resting value (mean ± standard deviation). To compare each mean to the control (DW) mean, the data were analyzed by one-way analysis of variance followed by Dunnett's multiple-comparison test. Tukey's honestly significant difference test was used to analyze the differences in LMSG secretion and BF changes following stimulation with various tastants.

The BF changes in the labial mucosa after the administration of DW, tastants, or autonomic agents are presented as a percentage of the baseline value recorded with no administration (mean ± standard deviation). To compare each mean to the control (DW) mean, the data were analyzed by one-way analysis of variance followed by Dunnett's multiple-comparison test.

The normality of the data was assessed using the Shapiro-Wilk test, and the correlation between the changes in LMSG secretion and the nearby BF was then statistically analyzed using Spearman's rank correlation. All statistical analyses were performed using SPSS 18.0 (SPSS Inc., Chicago, IL, USA). The criterion for significance was defined as *p* < 0.05.

## 3. Results

### 3.1. Changes in LMSG Secretion following Stimulation with Five Basic Tastes

**Figure 1:**
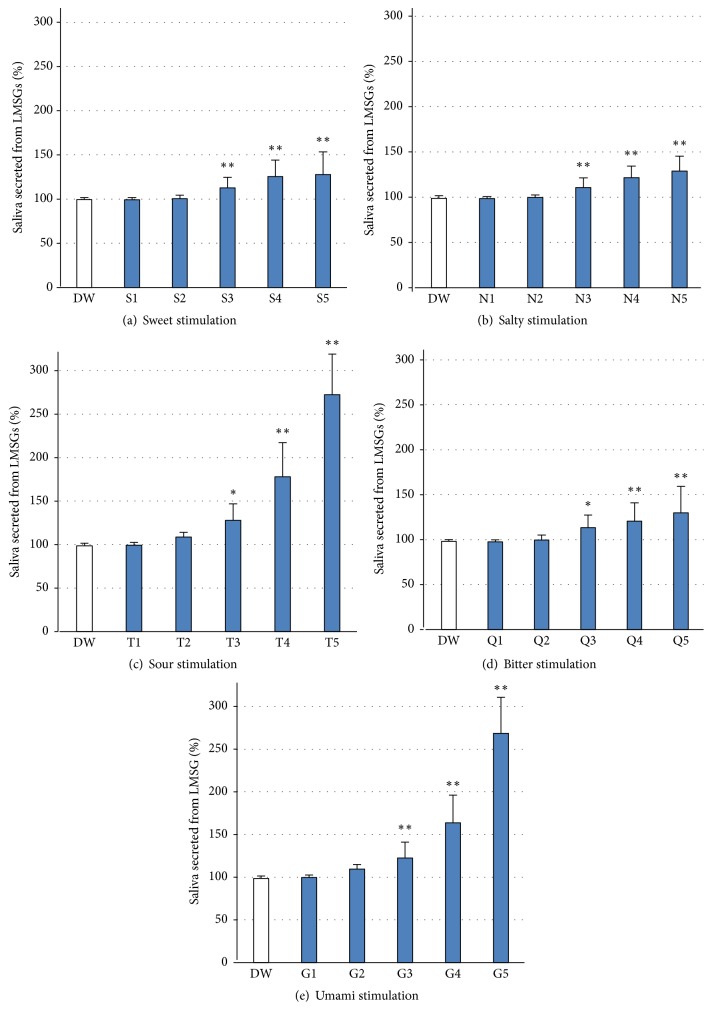
Changes in LMSG secretion following stimulation with five basic tastes. High concentrations (Nos. 3–5) of each of the five basic tastes (S, N, T, Q, and G) elicited a significant increase in LMSG secretion in human participants (*n* = 21), although lower concentrations (Nos. 1 and 2) of each solution caused no significant change. Ordinate: a percentage (%) of the resting saliva. ^*∗*^*p* < 0.05, ^*∗∗*^*p* < 0.0001.

Low concentrations of the five basic tastes (sweet, salty, sour, bitter, and umami) caused no significant changes in LMSG secretion; however, high concentrations (Nos. 3–5) of all tastes evoked significant increases in LMSG secretion (Figure 1).  Table 1 shows the detailed results.

As shown in Table 2, sour and umami tastes evoked significantly larger increases in LMSG secretion than did the other tastes (sweet, salty, and bitter) at high concentrations (Nos. 4 and 5), although low concentrations (Nos. 1–3) they did not.

### 3.2. Changes in LMSG Secretion following Stimulation with Mixed Umami Substances

**Figure 2:**
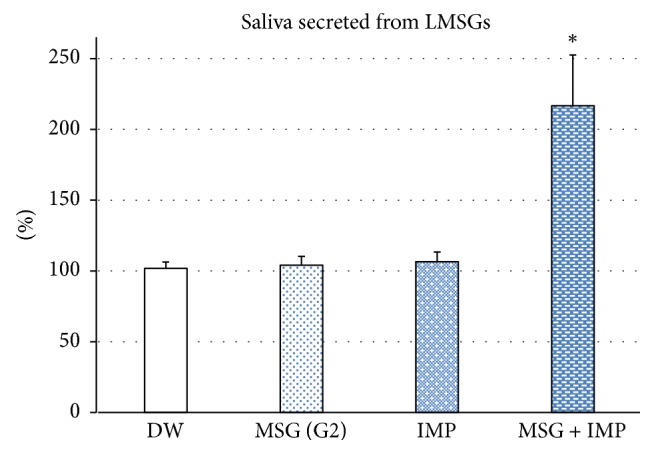
Changes in LMSG secretion following stimulation with umami substances. Neither MSG (G2) nor IMP elicited a significant change in LMSG secretion, while mixed umami substance (5 mM MSG + 5 mM IMP) caused a significant increase in LMSG secretion (*p* < 0.0001) (*n* = 10). ^*∗*^*p* < 0.0001. Ordinate: a percentage (%) of the resting saliva.

Mixed umami substances of 5 mM MSG and 5 mM IMP caused a significant increase in LMSG secretion (*p* < 0.0001), while each solution alone elicited no significant change (MSG G2: 104.1 ± 6.2, *p* = 0.985; IMP: 106.6 ± 6.8, *p* = 0.886) as compared with DW stimulation (101.8 ± 4.5) (Figure 2).

### 3.3. Relationship between LMSG and Nearby Lip BF following Taste Stimulation

**Figure 3:**
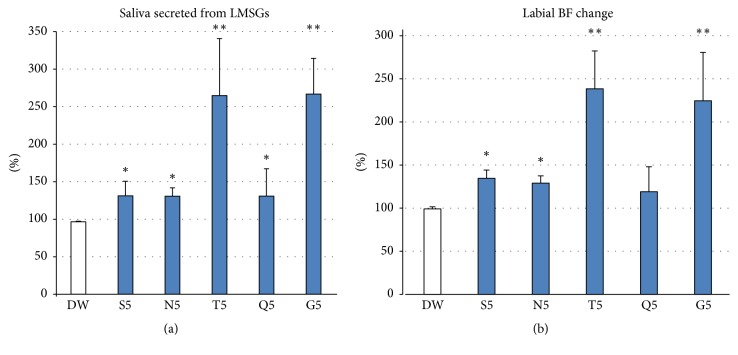
Changes in (a) LMSG secretion and (b) nearby BF change following stimulation with five basic tastes. (a) The highest concentration (No. 5) of each of the five basic tastes (S, N, T, Q, and G) evoked significant increases in MSG secretion (*n* = 14). (b) The same concentration of each of the basic tastes except bitter elicited a significant increase, but not a significant change, in labial mucosal BF (*n* = 14). Ordinate: a percentage (%) of (a) the resting saliva and (b) the baseline BF value. ^*∗*^*p* < 0.05, ^*∗∗*^*p* < 0.0001.

**Figure 4:**
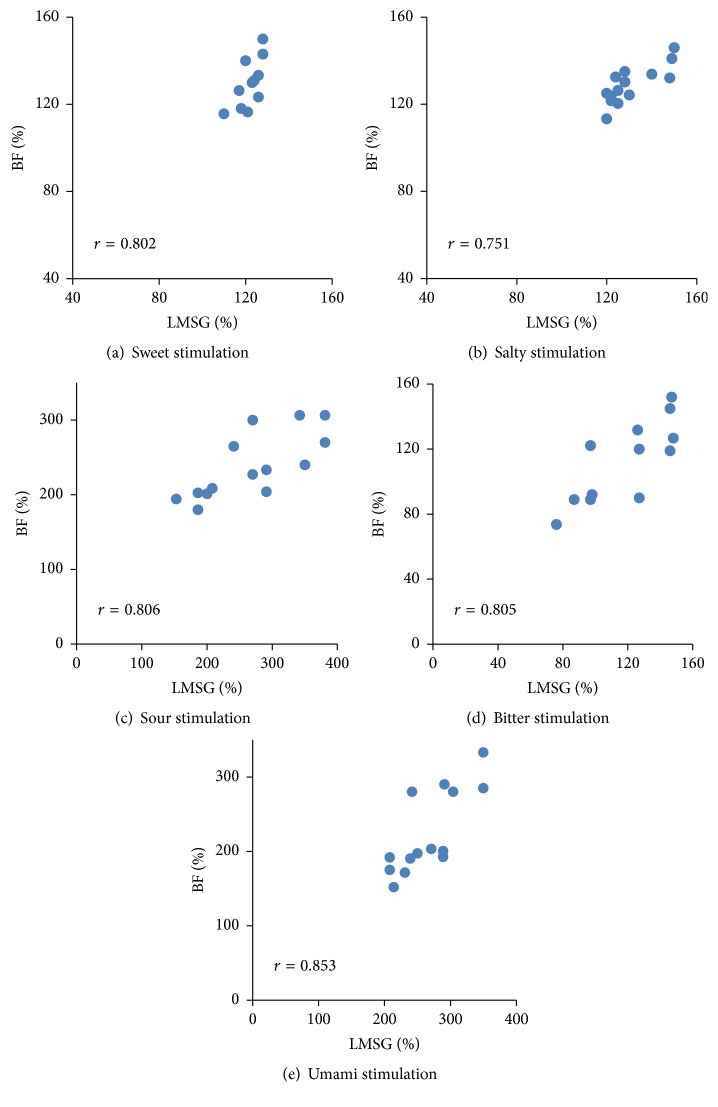
Relationship between LMSG secretion and BF response in lip following taste stimulation. Significant correlations were present between the amount of change in LMSG secretion and BF evoked by the highest concentration (No. 5) of each taste stimulus (*n* = 14). Ordinate: a percentage (%) of the baseline BF value; Abscissa: a percentage (%) of the resting LMSG saliva.

The highest concentration (No. 5) of each of the five basic tastes evoked a significant increase in LMSG secretion (sweet S5: 131.3 ± 19.3, *p* = 0.045; salty N5: 130.8 ± 11.1, *p* = 0.049; sour T5: 264.8 ± 76.4, *p* < 0.0001; bitter Q5: 131.0 ± 36.3, *p* = 0.048; umami: 266.8 ± 47.6, *p* < 0.0001) compared with DW stimulation (96.7 ± 0.8) (Figure 3(a)). All tastes except bitter evoked a significant increase in lip BF (sweet S5: 134.5 ± 9.5, *p* = 0.013; salty N5: 128.9 ± 8.6, *p* = 0.047; sour T5: 238.5 ± 43.8, *p* < 0.0001; umami G5: 224.4 ± 56.4, *p* < 0.0001) compared with DW stimulation (99.0 ± 2.5), while bitter did not elicit a significant change in BF (118.9 ± 29.1, *p* = 0.295) (Figure 3(b)). As shown in Tables 3 and 4, sour and umami tastes evoked significantly larger increases in both LMSG secretion and BF changes than did the other tastes (sweet, salty, and bitter). Some participants showed increases in both LMSG secretion and BF change in response to bitter taste, but others showed decreases in both LMSG secretion and BF change. Comparison of the changes in the same participants revealed a significant correlation between the amount of changes in salivation and BF in response to each taste stimulus (sweet: *r* = 0.802; salty: *r* = 0.751; sour: *r* = 0.806; bitter: *r* = 0.805; umami taste: *r* = 0.853) (Figure 4).

### 3.4. Changes in LMSG Secretion and Nearby Lip BF Change following Stimulation with Autonomic Agents

**Figure 5:**
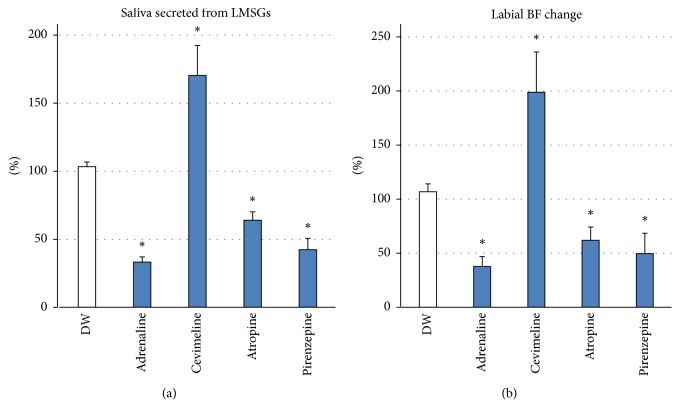
Changes in (a) LMSG secretion and (b) nearby BF change following stimulation with autonomic agents onto the lip. (a) Administration of cevimeline chloride caused a significant increase in LMSG secretion (*p* < 0.001), while adrenaline, atropine, and pirenzepine evoked significant decreases in LMSG secretion (*n* = 11). (b) Cevimeline chloride caused a significant increase in labial mucosal BF, while adrenaline, atropine, and pirenzepine elicited significant decreases (*n* = 11). Ordinate: a percentage (%) of the (a) resting saliva and (b) baseline BF. ^*∗*^*p* < 0.0001.

**Figure 6:**
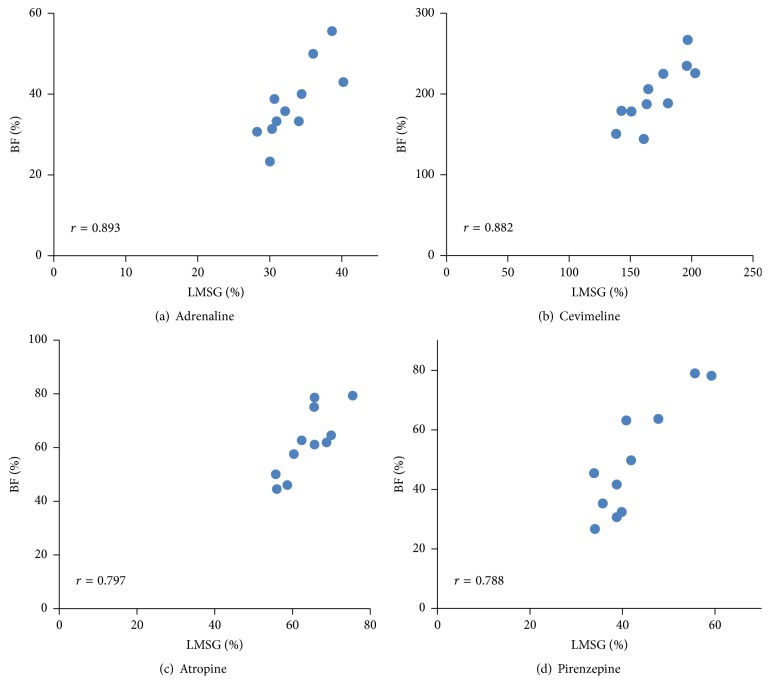
Relationship between LMSG secretion and BF response in lip following stimulation with autonomic agent. Significant correlations were present between the amounts of change in LMSG secretion and BF evoked by each autonomic agent in the same participant (*n* = 11). Ordinate: a percentage (%) of the baseline BF value; abscissa: a percentage (%) of the resting LMSG saliva.

Administration of cevimeline chloride (parasympathetic agonist) caused a significant increase in LMSG secretion (170.3 ± 22.1, *p* < 0.0001), while adrenaline (sympathetic agonist) (33.2 ± 3.8, *p* < 0.0001), atropine (parasympathetic inhibitor) (64.0 ± 6.1, *p* < 0.0001), and pirenzepine (parasympathetic antagonist) (42.3 ± 8.4, *p* < 0.0001) evoked a significant decrease in LMSG secretion compared with DW stimulation (103.4 ± 3.4) (Figure 5(a)). These changes induced by the different agents were consistent with those of nearby lip BF changes; that is, cevimeline chloride caused a significant increase in the BF (198.9 ± 37.1, *p* < 0.0001), while adrenaline (37.7 ± 9.2, *p* < 0.0001), atropine (61.9 ± 12.2, *p* < 0.0001), and pirenzepine (49.6 ± 18.8, *p* < 0.0001) elicited a significant decrease in the BF compared with DW stimulation (106.8 ± 7.2) (Figure 5(b)). Significant correlations were found between the amount of change in LMSG secretion and the BF for each autonomic agent in the same participant (adrenaline: *r* = 0.893; cevimeline: *r* = 0.882; atropine: *r* = 0.797; pirenzepine: *r* = 0.788) (Figure 6).

## 4. Discussion

### 4.1. Responses of Minor Salivary Gland Secretion to Stimulation with Five Different Tastes

The gustatory-salivary reflex (i.e., taste-initiated secretion of saliva) is important for tasting, masticating, and swallowing food. This vital reflex has been mainly studied in the saliva secreted from the major salivary glands or mixed saliva secreted from the major and minor salivary glands. Kerr [12] showed that the human major salivary flow response to citric acid, salt, and sucrose was 10, 7, and 4 times higher than the resting saliva response, respectively. Hodson and Linden [13] also demonstrated that the five basic taste qualities (sweet, salty, sour, bitter, and umami) induced the gustatory-salivary reflex in the parotid gland and that parotid salivary flow increased in a dose-dependent manner in response to umami taste (MSG).

Few reports have provided a detailed comparison of gustatory-salivary reflex salivation in response to the different taste stimuli in the minor salivary glands, except our preliminary report [14], because of the difficulty in measurement of the minute secretion volume from the minor salivary glands. In the present study, we used a newly developed method for measuring the LMSG flow rate [7] and demonstrated that (1) each of the five basic taste stimuli elicited a significant increase in saliva secreted from the LMSG in a dose-dependent manner, and (2) sour and umami tastes elicited significantly larger increases in LMSG secretion than did sweet, salty, or bitter. These results are consistent with previous reports demonstrating the major salivary flow response [13, 15, 16].

Allen [17] reported a correlation between gustatory-salivary reflex salivation in the parotid gland and the intensity of the taste stimulus. Therefore, the taste intensity of each different taste quality must be equivalent to compare the differences in the amount of saliva produced by the gustatory-salivary reflex. In the present study, each different taste quality solution, including umami, was administered at five different intensities (Nos. 1–5) based on a previous study that established the cumulative distribution of each tastant [10, 11]. For example, the specific taste quality of the No. 2 concentration of each taste solution can be recognized by 50% of participants. Thus, using the same number of taste test solutions, it becomes possible to supply an equal intensity of taste perception in spite of the differences in taste quality.

### 4.2. Responses of LMSG Secretion to Stimulation with Mixed Umami Substance

Mixed umami solution containing MSG and IMP caused a significant increase in LMSG secretion, whereas stimulation with MSG or IMP alone did not increase LMSG secretion at these concentrations. The synergism of umami tastes between MSG and guanylate was first reported by Kuninaka [18, 19], and Yamaguchi and Ninomiya [9] indicated that the detection threshold of umami taste perception of MSG was markedly lower in the presence of IMP. A recent electrophysiological study involving mice demonstrated the occurrence of marked enhancement of the glossopharyngeal nerve responses to MSG by the addition of guanylate [20, 21]. This is in line with our result on the synergism of umami tastes when the posterior tongue is stimulated by a mixture of MSG and another umami substance (e.g., IMP). It has been suggested that the human taste receptor, a T1R1 + T1R3 heterodimer, induces potentiation of the synergism between MSG and IMP. A recent study suggested the existence of separate binding sites for MSG and IMP within the same T1R1 Venus flytrap domain, which is important for umami taste synergism [22, 23]. In T1R1-knockout mice, the synergism between MSG and IMP is considerably reduced in the anterior tongue [24]. Thus, the umami taste has a quite distinguished synergistic effect exhibited by no other taste quality. We demonstrated that the synergistic effect of umami not only showed sensory perception but also evoked the gustatory-salivary reflex in the LMSGs. This synergistic effect has also been shown to be elicited not only between MSG and IMP but also between MSG and other nucleotides of guanylate [18, 19]. Therefore, further studies of the effect of different mixtures of MSG and other nucleotides on reflex secretion in the LMSGs are needed.

Umami has another specific characteristic, that is, its residual aftertaste, which differs from other taste qualities [25]. In a preliminary study, we examined the time course of the salivary flow of LMSG secretion in response to the five basic tastes and found that the umami taste evoked a long-lasting increase in LMSG, whereas sour taste evoked a prominent increase in the LMSG flow that immediately diminished [14]. It seems likely that these long-lasting effects on LMSG secretion incidental to the umami taste are due to the residual aftertaste. The synergism and residual aftertaste of the umami taste may be beneficial for patients with dry mouth based on our previous finding that xerostomia is more strongly related to the LMSG flow than the major salivary gland flow [8].

### 4.3. Relationship between LMSG Secretion and Nearby Lip BF Change following Taste Stimulation

The salivary glands are supplied by a dense capillary network equivalent to that of the heart; thus, vasodilatation of these capillaries surrounding the salivary glands might be necessary to ensure that large volumes of saliva are produced by the secretory cells [26, 27]. We considered that the circulation surrounding the LMSGs is closely related to the LMSG secretory system. Consequently, we examined the nearby lip BF where the LMSG secretion measurement was performed using laser Doppler flowmetry. Our measurement of the BF included the labial glandular BF because laser Doppler flowmetry can measure the erythrocyte flux through an approximately 1 mm^3^ volume of the capillary bed without touching the tissues [28]. This can be accomplished because the LMSGs densely exit via the superficial oral mucosa, which is very thin. We demonstrated that stimulation with all tastes except bitter caused an increase in the nearby lip BF consistent with the increase in the LMSG secretion. In addition, sour and umami tastes induced prominent increases in the BF in the same manner as the LMSG secretion. Taste stimulation evoked no BP changes, indicating that vasodilation in the stimulated area was induced.

Interestingly, we observed a correlation between the rate of changes in the BF and the LMSG response to each taste stimulus in different participants (Figure 4). As shown in Figure 4, sweet, salty, sour, and umami tastes evoked correlated increases in the BF and LMSG secretion (>100% in the figure) in all participants; however, bitter caused a correlated decrease in the BF and LMSG secretion (<100% in the figure) in some participants. Thus, bitter only evoked a BF decrease in some participants. A previous study showed that the BF in the orofacial area is uniquely controlled by a double autonomic system; that is, vasoconstriction mediated via sympathetic nerve fibers and vasodilation mediated via parasympathetic efferent nerve fibers [29]. Bitter taste can evoke both sympathetically induced reflexive vasoconstriction and parasympathetically mediated vasodilation, while the other tastes prominently induce reflex vasodilation. In this respect, the hedonic dimension to the taste reportedly plays various roles in the many taste-mediated whole-body responses. Interestingly, an unpleasant bitter taste can reportedly induce sympathetically mediated physiological changes in skin BF and skin temperature, instantaneous heart rate, and skin potential and skin resistance much more strongly than other taste qualities (sweet, salty, and sour) [30]. Additionally, pleasant stimuli were found to elicit approach and acceptance, whereas unpleasant stimuli induced avoidance and rejection, thus determining taste preferences and aversions [30]. Although we did not record the participants' liking of each tastant in this experiment, some participants indeed hated the bitter taste. Such individuals may show stronger decreases in LMSG secretion and the BF as a sympathetic effect. Further studies are needed to clarify the role of unpleasant taste sensations in the control of taste-mediated responses related to food rejection.

Overall, our results indicate that the BF change surrounding the LMSGs is an important factor in the salivary secretory system in the LMSGs.

### 4.4. LMSG Secretion and Nearby Lip BF Changes Mediated by the Autonomic Nervous System

Salivary secretion is controlled by the parasympathetic and sympathetic autonomic nervous systems [31]. In the human parotid gland, the gustatory-salivary reflex involves the activity of both types of autonomic nerves, while the masticatory-salivary reflex preferentially activates the parasympathetic nerves [32]. Mobilization of the intracellular messenger calcium by stimulation of muscarinic receptors (M1, M3) is associated with fluid secretion, particularly large volumes in response to muscarinic agonists, via exocytosis in the rat parotid gland [33]. However, the neural regulation of the gustatory-salivary reflex in human LMSGs remains unknown. In the present study, application of cevimeline hydrochloride hydrate (an agonist of the muscarinic M3 receptor) onto the lip elicited an increase in LMSG secretion. Furthermore, pirenzepine (an antagonist of the muscarinic M1 receptor) and atropine (a competitive inhibitor of the muscarinic acetylcholine receptor) elicited a decrease in LMSG secretion. Thus, we conclude that muscarinic receptors (M1, M3) are engaged in human LMSG secretion. However, the application of adrenaline (an agonist of *α* and *β* adrenergic receptors) certainly decreased LMSG secretion. This phenomenon differs from that described in a report on sympathetic nerve-induced secretion by the parotid gland, suggesting that the human LMSGs may lack sympathetic secretion. This idea is supported by a histochemical study indicating that few adrenergic nerves have been identified in the human LMSGs [34].

Nervous control of the orofacial BF is regulated by both the parasympathetic and sympathetic autonomic nervous systems [29]. In the cat, labial BF is controlled by two groups of parasympathetic fibers (the facial and glossopharyngeal nerves) for vasodilatation [29] and by sympathetic *α*-adrenergic fibers for vasoconstriction [35]. We also examined the neural regulation of the BF in the human labial mucosa because salivary secretion appears to be related to the nearby BF, as mentioned above. In our pharmacological analysis, application of cevimeline hydrochloride hydrate (an agonist of the muscarinic M3 receptor) elicited a prominent increase in the BF without a change in the BP, and pirenzepine (an antagonist of the muscarinic M1 receptor) and atropine (a competitive inhibitor of the muscarinic acetylcholine receptor) elicited a significant decrease in the BF without a change in the BP, indicating that muscarinic receptors (M1, M3) are engaged in vasodilatation in the human labial mucosal tissues surrounding the LMSGs. Furthermore, adrenaline (an agonist of *α* and *β* adrenergic receptors) elicited a significant decrease in the nearby BF without a change in the BP, indicating that *α*-adrenergic receptors are involved in vasoconstriction in this region. Interestingly, correlations were found between the dynamics of the saliva secreted from the LMSGs and the nearby lip BF changes in response to each chemical agent in the same participants (Figure 6), although vascular responses monitored by laser Doppler flowmetry should include not only the labial glandular BF but also the mucosal capillary BF. These results show that parasympathetic activation can simultaneously increase the salivary secretion from the LMSGs and induce vasodilatation in the mucosal tissues surrounding the LMSGs. Conversely, decreases in the saliva secreted by the LMSGs may be caused by a decrease in BF incidental to the vasoconstriction because the human LMSGs possibly lack sympathetic secretion, as discussed above [34]. Thus, we consider that LMSG secretion is strongly influenced by the nearby BF. Further detailed studies are necessary to clarify the effects of the relationship between LMSG secretion and nearby BF changes on the autonomic nervous system.

The present study has shown that each of the five basic taste sensations can induce human LMSG secretion. This LMSG secretion is an autonomic nervous system-induced reflex that spontaneously arises at meals and may be beneficial to various functions of eating, such as smooth chewing and formation of a food bolus. Moreover, LMSG secretion provides lubrication and protection of the oral mucosa because the LMSG secretions contain high concentrations of protective substances such as mucin and immunoglobulin A. This LMSG secretion induced by taste substances contained in food at meals would thus be beneficial for maintaining oral health.

## 5. Conclusions

Taste stimulation can cause a gustatory-reflex secretion in the human LMSGs. In particular, sour and umami tastes cause larger increases in LMSG secretion than do other tastes. Umami has a synergistic effect on the LMSG secretion reflex. Parasympathetic regulation is involved in the gustatory-salivary reflex in the LMSGs in association with the changes in BF near the LMSGs.

## Figures and Tables

**Table 1:** Changes in LMSG secretion induced by DW or tastants.

	DW	1	2	3	4	5
		S (sweet)				
% change	99.5 ± 2.3	99.1 ± 2.6	100.4 ± 4.1	112.8 ± 8.7	125.5 ± 16.6	126.4 ± 25.5
*p* values	-	0.999	0.998	<**0.000**1^**∗∗**^	<**0.000**1^**∗∗**^	<**0.000**1^**∗∗**^

		N (salty)				
% change	100.6 ± 3.0	98.5 ± 2.2	108.9 ± 8.7	114.1 ± 12.5	118.7 ± 14.1	119.8 ± 16.6
*p* values	-	0.999	0.966	<**0.000**1^**∗∗**^	<**0.000**1^**∗∗**^	<**0.000**1^**∗∗**^

		T (sour)				
% change	98.7 ± 2.8	99.3 ± 3.2	111.1 ± 7.2	134.2 ± 15.8	174.2 ± 35.7	272.4 ± 42.5
*p* values	-	0.999	0.696	0.007^**∗**^	<**0.000**1^**∗∗**^	<**0.000**1^**∗∗**^

		Q (bitter)				
% change	104.0 ± 1.9	97.7 ± 1.9	105.1 ± 9.7	115.1 ± 18.9	119.0 ± 26.8	121.1 ± 34.3
*p* values	-	0.999	0.994	0.001^**∗**^	<**0.000**1^**∗∗**^	<**0.000**1^**∗∗**^

		G (ummai)				
% change	98.5 ± 2.9	99.6 ± 3.1	109.4 ± 5.4	122.5 ± 18.6	163.7 ± 32.5	268.6 ± 42.1
*p* values	-	0.999	0.998	<**0.000**1^**∗∗**^	<**0.000**1^**∗∗**^	<**0.000**1^**∗∗**^

Statistical differences were analysed by one-way ANOVA and Dunnett's multiple-comparison test (^*∗*^*p* < 0.05, ^*∗∗*^*p* < 0.001); % change indicates a percentage of the resting value (mean ± standard deviation); *n* = 21.

**Table 2:** Increases in LMSG secretion induced by high concentration of tastants (No. 4 and 5).

		S (sweet)	N (salty)	T (sour)	Q (bitter)	G (umami)
No. 4	S (sweet)	-	0.977	<0.001^*∗*^	0.949	<0.001^*∗*^
	N (salty)		-	<0.001^*∗*^	1.000	<0.001^*∗*^
	T (sour)			-	<0.001^*∗*^	0.21
	Q (bitter)				-	<0.001^*∗*^
	G (umami)					-

No. 5	S (sweet)	-	1.000	<0.001^*∗*^	1.000	<0.001^*∗*^
	N (salty)		-	<0.001^*∗*^	1.000	<0.001^*∗*^
	T (sour)			-	<0.001^*∗*^	0.999
	Q (bitter)				-	<0.001^*∗*^
	G (umami)					-

Statistical differences were analysed by one-way ANOVA and Tukey's honestly significant difference test; numerical value means *p* value between one tastant and another: *∗* (asterisk) means significant; *n* = 21.

**Table 3:** Increases in LMSG secretion induced by tastant of No. 5.

		S (sweet)	N (salty)	T (sour)	Q (bitter)	G (umami)
No. 5	S (sweet)	-	0.999	<0.001^*∗*^	0.826	<0.001^*∗*^
	N (salty)		-	<0.001^*∗*^	0.697	<0.001^*∗*^
	T (sour)			-	<0.001^*∗*^	0.967
	Q (bitter)				-	<0.001^*∗*^
	G (ummai)					-

Statistical differences were analysed by one-way ANOVA and Tukey's honestly significant difference test; numerical value means *p* value between one tastant and another: *∗* (asterisk) means significant; *n* = 14.

**Table 4:** Increases in BF change induced by tastant of No. 5.

		S (sweet)	N (salty)	T (sour)	Q (bitter)	G (umami)
No. 5	S (sweet)	-	0.991	<0.001^*∗*^	0.717	<0.001^*∗*^
	N (salty)		-	<0.001^*∗*^	0.926	<0.001^*∗*^
	T (sour)			-	<0.001^*∗*^	0.791
	Q (bitter)				-	<0.001^*∗*^
	G (ummai)					-

Statistical differences were analysed by one-way ANOVA and Tukey's honestly significant difference test; numerical value means *p* value between one tastant and another: *∗* (asterisk) means significant; *n* = 14.
